# Maternal pelvic dimensions and neonatal size: Implications for growth plasticity in early life as adaptation

**DOI:** 10.1093/emph/eox016

**Published:** 2018-02-05

**Authors:** Jonathan C K Wells, José N Figueiroa, Joao G Alves

**Affiliations:** 1 Population, Policy and Practice Programme, Childhood Nutrition Research Centre, UCL Great Ormond Street Institute of Child Health, 30 Guilford Street, London WC1N 1EH, UK; 2 Department of Pediatrics and Statistics Unit, Faculdade Pernambucana de Saúde (FPS), Medical School, Instituto de Medicina Integral Professor Fernando Figueira (IMIP), Rua dos Coelhos 300, Boa Vista, Recife, PE Brazil CEP 52050-080, Brazil

**Keywords:** developmental plasticity, maternal pelvis, fetal growth, birth weight, adaptation

## Abstract

Patterns of fetal growth predict non-communicable disease risk in adult life, but fetal growth variability appears to have a relatively weak association with maternal nutritional dynamics during pregnancy. This challenges the interpretation of fetal growth variability as ‘adaptation’. We hypothesized that associations of maternal size and nutritional status with neonatal size are mediated by the dimensions of the maternal pelvis. We analysed data on maternal height, body mass index (BMI) and pelvic dimensions (conjugate, inter-spinous and inter-cristal diameters) and neonatal gestational age, weight, length, thorax girth and head girth (*n* = 224). Multiple regression analysis was used to identify independent maternal predictors of neonatal size, and the mediating role of neonatal head girth in these associations. Pelvic dimensions displaced maternal BMI as a predictor of birth weight, explaining 11.6% of the variance. Maternal conjugate and inter-spinous diameters predicted neonatal length, thorax girth and head girth, whereas inter-cristal diameter only predicted neonatal length. Associations of pelvic dimensions with birth length, but not birth weight, were mediated by neonatal head girth. Pelvic dimensions predicted neonatal size better than maternal BMI, and these associations were mostly independent of maternal height. Sensitivity of fetal growth to pelvic dimensions reduces the risk of cephalo-pelvic disproportion, potentially a strong selective pressure during secular trends in height. Selection on fetal adaptation to relatively inflexible components of maternal phenotype, rather than directly to external ecological conditions, may help explain high levels of growth plasticity during late fetal life and early infancy.

## INTRODUCTION

There is compelling evidence that growth patterns in early life predict diverse components of health or disease risk in later life, as summarized in the ‘developmental origins of adult health disease’ (DOHaD) hypothesis [1]. Classic studies of rodents in the 1960 s identified ‘sensitive periods’ or ‘critical windows’ during early development, during which growth patterns generated a long-term impact on later body size and composition [2]. The specific role of nutrition in these effects was demonstrated experimentally in animals, by administering low-protein or low-energy diets during pregnancy [3, 4]. In humans, observational studies from the 1990 s onwards have likewise linked variability in size at birth with the risk of non-communicable diseases (NCDs), including stroke, hypertension, type II diabetes and cardiovascular disease [1, 5].

Initially, most attention was directed to the high NCD risk among those of low birth weight (<2500 g), seemingly implicating ‘fetal under-nutrition’ as the primary pathophysiological mechanism [6]. This interpretation received support from long-term follow-up studies of those exposed *in utero* to maternal famine during the Dutch Hunger Winter, in 1944–45 [7]. Notably, however, similar analysis of those gestated during the Leningrad siege (1941–44) failed to replicate the Dutch findings [8]. A crucial difference was that the Dutch experienced only a brief and well-defined period of starvation, followed by rapid restoration of food supplies, whereas Leningrad experienced severe famine for several years. Dutch fetuses exposed to maternal famine are likely to have experienced catch-up growth after birth, an established independent risk factor for NCDs [9].

Exactly how maternal nutrition during pregnancy shapes NCD risk in the offspring is also controversial from other perspectives. First, the association between birth weight and later NCD risk holds across the entire spectrum of birth weight, so that each additional increment of birth weight is associated with lower risk [5, 10]. On this basis, overt fetal *malnutrition* does not appear to be the key mechanism. Second, circulating maternal nutrient levels during pregnancy show negligible association with birth weight [11, 12], though a few studies have linked specific factors such as maternal glycaemic load or fish intake during pregnancy with birth weight [13, 14]. Maternal protein-energy supplementation during pregnancy typically results in relatively modest birth weight increments [15], though increases of 200–300 g have been reported among the most malnourished mothers [16, 17]. Collectively, these studies suggest that maternal diet during pregnancy can impact fetal growth, but that the magnitude of the effect tends to be modest.

More generally, the majority of evidence linking early plasticity with later NCD risk relates not to maternal or fetal nutrition, rather to *early growth variability*. Moreover, recent large cohort studies indicate that among adults living a healthy lifestyle, there is little association of birth weight variability with adult NCD risk, whereas among adults with unhealthy lifestyle (obese, sedentary, unhealthy diet, smoking) birth weight is inversely associated with NCD risk [18, 19]. These data fit a ‘capacity-load’ model of NCD aetiology, in which the long-term capacity for homeostasis develops in fetal life and infancy in association with the magnitude of early growth, and NCD risk becomes elevated if those with low capacity subsequently acquire a high metabolic load [20, 21]. In high-income populations, where low birth weight is often followed by catch-up growth [9], there may be an inherent tendency for those with low capacity to acquire elevated load, elevating their NCD risk.

Natural selection broadly favours larger neonates, due to their better survival in post-natal life [22, 23], though very high birth weights contradict this trend and indicate excess adiposity. The fact that median birth weight is substantially lower than the level at which survival is maximized indicates a tendency for mothers to constrain fetal growth, which may promote maternal genetic fitness over that of each individual offspring [23, 24]. Classic studies by Ounsted and colleagues indicated inter-generational transmission of a mechanism constraining fetal growth through the female line, indicating that paternal effects were negligible when maternal constraint is severe [25, 26]. While genetic factors might contribute, these analyses also indicated the involvement of non-genetic mechanisms.

**Figure 1.**
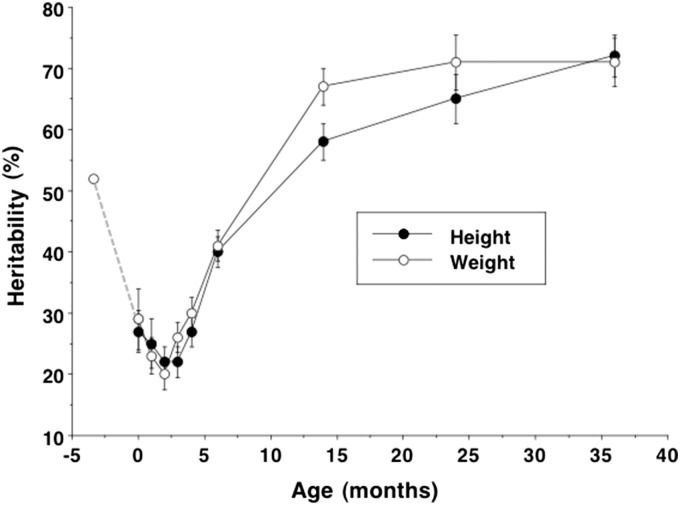
Estimates of heritability in weight and length/height in the Netherlands Twin Register study, with data from another study of late pregnancy added. Heritability of weight declines from ∼50% at 25 weeks gestation to ∼30% at birth, then increases to ∼70% by 36 months. The post-natal pattern for length is very similar. Data from Gielen *et al.* [29] and Mook-Kanamori *et al.* [30]. Reprinted with permission from Ref. [39]

Of relevance here, recent studies indicate a striking developmental pattern in the heritability in growth. Twin studies show that the heritability of adult height approaches ∼90% [27], contrasting markedly with that for birth weight of ∼30% [28]. However, both before and after birth, the heritability of growth is higher, as demonstrated in Fig. 1 [29, 30]. There is a profound dip in heritability around the time of birth, of which the inevitable counterpart is that phenotype is more plastic during late fetal life and early infancy. A key question is, why should plasticity increase at such a vulnerable stage of the life-course?

One evolutionary perspective on developmental plasticity is that the developing fetus seeks information about *external* ecological conditions, in order to develop an appropriate phenotype in anticipation of similar conditions in adulthood. According to this ‘predictive adaptive response’ hypothesis [31], NCDs develop when the environment changes between fetal life and adulthood, resulting in the organism being ‘mismatched’ to its adult environment. Two challenges for this hypothesis are first that maternal physiology buffers the fetus substantially from ecological stresses [32], and second that those gestated during famines have the lowest rates of survival or reproduction if encountering famine again in adult life [33], indicating no adaptive benefits of such hypothesized anticipatory matching.

An alternative perspective assumes that the growth trajectory of the fetus is imprinted by maternal phenotype, and that offspring must deal with the consequences of this imprint by adapting their subsequent developmental trajectory [21, 34]. In South Asian women, for example, the level of maternal investment in early life, proxied by their birth weight, predicted the timing of puberty, adult height, fatness and NCD risk [35]. Lower maternal investment predicted a faster life history trajectory, favouring reproduction over growth and metabolic health.

Maternal body mass index (BMI) is an established predictor of birth weight [36], but the underlying mechanisms require further investigation, since BMI is correlated with numerous aspects of body composition and metabolism. Several studies have reported stronger associations of maternal lean mass than fat mass with offspring birth weight [36, 37], but the underlying mechanism still requires elucidation. One small study linked maternal protein turnover, rather than dietary protein intake, with length but not weight of the neonate [38]. While lean mass encompasses various aspects of metabolism, it may also index components of physique that impact fetal growth potential.

In this context, the size of the maternal pelvis could represent an important constraint on fetal growth. The natural need for the fetus to pass through the pelvis during the birth process is predicted to suppress genetic influence on growth around the time of birth [39]. Upward and downward secular trends in adult height are now well established–positive, in association with improved living standards [40], or negative, such as following the origins of agriculture [41]. Although the evidence is sparse, several studies have reported that the dimensions of the maternal pelvis scale with stature [42, 43] though other studies failed to find such a relationship [44]. Furthermore, a secular increase in female stature was associated with a secular increase in pelvic dimensions [45]. We have hypothesized previously that secular trends in maternal size must inevitably impact the ease of delivery, and hence have constituted a major selective pressure on fetal growth patterns [46].

Surprisingly, however, the notion that maternal pelvic dimensions shape growth trajectory of the offspring has received relatively little attention in the DOHaD field. Flattened maternal pelvises were associated with subtle changes in the ratio of neonatal length to head girth in the offspring, which in turn predicted an elevated risk of stroke in adult life [47]. In India, smaller maternal pelvic dimensions were similarly associated with lower arterial compliance in the adult offspring [48]. A more indirect mechanism could be reduced pelvic vasculature in mothers of smaller size, reducing blood supply to the fetus [49]. However, potential broader links between maternal pelvic dimensions and offspring growth patterns require further investigation.

In 1903, Lane suggested that the size of the maternal pelvis must inevitably constrain fetal growth: ‘The child grows *in utero* in such a manner and at such a rate that at full term his size is proportional to that of the mother’s pelvis through which he has to pass in order to be born’ [50]. Within 4 years, his study had been replicated but his results and conclusion directly refuted [51]. More recent studies have produced inconsistent evidence regarding whether pelvic measurements of mothers can predict dystocia, obstructed labour and other birth complications on an individual basis [52, 53]. Whilst various components of maternal body size, proportions or composition (height, leg length, lean mass) are established predictors of birth weight [36, 54, 55], whether the pelvis might mediate these associations remains untested.

We therefore investigated whether pelvic dimensions of the mother predicted birth size of the offspring. We focused on two maternal traits–height, representing completed growth and hence potentially carrying an imprint of nutritional circumstances during the mother’s own development, and BMI, a composite index of nutritional status. We hypothesized that pelvic dimensions would mediate associations of maternal height and BMI with neonatal body size. We re-analysed data from a study in Recife, Brazil which had previously reported reduced pelvic dimensions in adolescent mothers compared to those reproducing post-adolescence [56]. We restricted our new analyses to the post-adolescent mothers, and explored a wider range of components of neonatal size.

## METHODS

We analysed data on adult women who gave birth at the Instituto de Medicina Integral Professor Fernando Figueira (IMIP) between October and December 2010 [56]. Written consent was obtained from each individual, and ethical approval was granted by the IMIP Ethics Committee.

Since previous work in this cohort has already shown that adolescent mothers have smaller pelvic dimensions [56], the inclusion criteria for this analysis were women aged 19–45 years with low-risk term pregnancy, producing singleton offspring. Exclusion criteria were maternal pre-eclampsia, gestational diabetes, type 1 or type 2 diabetes mellitus, or mothers who reported smoking, alcohol intake or drug use during pregnancy. Newborns were also excluded if they had congenital infections, malformations or genetic syndromes. Maternal age (years) and gestational age (weeks) were recorded. Birth order of the offspring was categorized into three groups, first-born, second-born or third^+^-born.

Clinic pelvimetry was carried out by two trained researchers using a Collin pelvimeter, with duplicate measurements taken. External pelvic measurements included the conjugate diameter (distance between pubic symphysis and spinous process of the 5th lumbar vertebra), the intercristal diameter (maximum width across iliac crests), and the interspinous diameter (distance between anterior superior iliac spines). Maternal weight and height were measured and BMI (BMI, kg/m^2^) calculated. In the newborn, anthropometric measurements of weight, length and girths of the thorax and head were obtained. All maternal and infant measurements were taken within 24 h of delivery.

Correlation analysis was used to investigate crude associations among maternal traits, among infant traits, and between maternal and infant traits. Potential differences in maternal or offspring traits in association with birth order and offspring sex were tested by ANOVA, with birth order models correcting for multiple comparisons using the Bonferroni method.

Multiple regression analysis was then used to investigate associations of maternal body size (height) or nutritional status (BMI) with neonatal size, and the extent to which such associations were mediated by maternal pelvic dimensions. Initial models included only single pelvic dimensions, while the final model included all three dimensions. Additional models tested whether associations between maternal pelvic dimensions and neonatal length or weight were mediated by neonatal head girth. All models were adjusted where relevant for birth order and offspring sex.

## RESULTS

A description of the sample of 224 mother-infant dyads is given in Table 1. Average maternal age was 26.6 years, ranging from 19 to 42 years, average BMI was 26.2 kg/m^2^, ranging from 16 to 42 kg/m^2^ and average birth weight was 3150 g, ranging from 2000 to 4350 g. There were 116 male and 108 female offspring.

**Table 1.** Description of maternal and infant variables

Trait	Mean	SD	Range
Mother			
Age (*y*)	26.6	5.5	19–42
Weight (kg)	65.5	10.6	40–100
Height (cm)	157.9	6.1	144–175
BMI (kg/m^2^)	26.2	3.9	16–42
Conjugate diameter (cm)	24.0	2.2	18–31
Inter-spinous diameter (cm)	20.2	2.5	14–26
Inter-cristal diameter (cm)	20.5	2.4	15–29
Neonate			
Gestational age (weeks)	39.0	1.2	37–42
Birth weight (g)	3150	488	2000–4350
Birth length (cm)	48.1	2.2	41–54
Birth thorax girth (cm)	32.7	2.1	26–45
Birth head girth (cm)	34.5	1.7	31–46

SD, standard deviation.

Table 2 reports correlation coefficients across maternal and infant traits. Maternal height was weakly correlated with pelvic dimensions (*r* = 0.06–0.21), while BMI showed higher correlations (*r* = 0.47–0.68). The pelvic dimensions were inter-correlated with coefficients of 0.49–0.67. Maternal height was correlated with birth length and weight, but not thorax or head girth, while maternal BMI was only correlated with birth weight. Among infants, birth size was positively correlated with gestational age and every component of birth size was correlated with all others (*r* = 0.56–0.75). There was no significant association by ANOVA of birth order with maternal height, BMI or pelvic dimensions, nor with any component of neonatal size. Second-born and third^+^-born offspring were 133 g (95% CI–68 335) and 97 g (95% CI–100 293) heavier, respectively, compared to first-born offspring. Offspring sex was not associated with any neonatal or maternal trait, and was not considered in subsequent regression models.

**Table 2.** Correlations among maternal traits and neonatal size

	Maternal trait				Neonatal trait			
	Conjugate	Inter-spinous	Inter-cristal	Gestational age	Weight	Length	Thorax	Head girth
Maternal trait								
Height	0.21 **	0.06	0.16 *	0.16 *	0.15 *	0.19 **	0.08	0.09
BMI	0.56 ***	0.47 ***	0.68 ***	0.02	0.16 *	–0.03	0.06	0.06
Conjugate	––	0.67 ***	0.54 ***	0.06	0.24 ***	0.15 *	0.19**	0.18 *
Inter-spinous	––	––	0.49 ***	0.13	0.31 ***	0.17 *	0.20**	0.22 **
Inter-cristal	––	––	––	0.11	0.29 ***	0.11	0.13	0.15 *
Neonatal trait								
Gestational age					0.36 ***	0.32 ***	0.25 ***	0.29 ***
Weight						0.68 ***	0.75 ***	0.65 ***
Length							0.57 ***	0.56 ***
Thorax girth								0.69 ***

Shading indicates significance *P* < 0.05.

* *P < 0*.05.

** *P ≤ 0*.005.

*** *P ≤ 0*.0001.

Table 3 provides multiple regression models for the prediction of neonatal size. Second-born status was significant in some models for birth weight and head girth, while third^+^-born status was significant only in the birth weight model that included all three pelvic dimensions. Taking any such birth order associations into account, maternal BMI predicted birth weight, but this association disappeared if any pelvic dimension was added to the model. In the combined model, inter-spinous and inter-cristal diameters remained independent predictors of birth weight, and together they explained 11.6% of the variance in birth weight. For birth length, maternal BMI was negatively associated, but not significantly so. When pelvic dimensions were incorporated, each was an independent predictor of birth length, and the inverse association of maternal BMI was now significant or nearly so. In the combined model, no individual pelvic dimension predicted birth length, but the negative association with maternal BMI remained significant. For neonatal thorax and head girth, the conjugate and inter-spinous diameter were predictors in simple models, but in the combined model none was significant.

**Table 3.** Multiple regression models of offspring size at birth on maternal BMI and pelvic dimensions

Predictor	Neonatal weight (g)				Neonatal length (cm)				Neonatal thorax girth (cm)				Neonatal head girth (cm)			
	Beta	SE	*P*	*r*^2^	Beta	SE	*P*	*r*^2^	Beta	SE	*P*	*r*^2^	Beta	SE	*P*	*r*^2^
Constant	–4307	1139	<0.0001	0.16	27.3	5.4	<0.0001	0.10	13.9	5.2	0.008	0.06	16.9	4.2	<0.0001	0.08
Gestational age (*w*)	146.7	24.0	<0.0001		0.58	0.11	<0.0001		0.42	0.11	<0.0001		0.40	0.09	<0.0001	
Maternal BMI (kg/m^2^)	523.0	206.1	0.012		–0.55	0.98	0.5		0.79	0.95	0.4		0.66	0.76	0.3	
Constant	–4054	1124	<0.0001	0.19	28.6	5.3	<0.0001	0.13	15.2	5.1	0.004	0.08	17.0	4.1	<0.0001	0.11
Gestational age (*w*)	143.9	23.6	<0.0001		0.56	0.11	<0.0001		0.40	0.11	<0.0001		0.40	0.09	<0.0001	
Maternal BMI (kg/m^2^)	127.4	244.3	0.6		–2.43	1.16	0.037		–0.94	1.13	0.4		–0.68	0.90	0.4	
Conjugate (cm)	47.6	16.4	0.004		0.23	0.08	0.004		0.21	0.08	0.006		0.16	0.06	0.009	
Constant	–3537	1121	0.002	0.22	29.9	5.4	<0.0001	0.12	16.4	5.2	0.002	0.08	18.3	4.2	<0.0001	0.12
Gestational age (*w*)	135.4	23.4	<0.0001		0.54	0.11	<0.0001		0.38	0.11	0.001		0.38	0.09	<0.0001	
Maternal BMI (kg/m^2^)	98.8	226.4	0.6		–0.91	1.09	0.082		–0.46	1.06	0.6		–0.56	0.84	0.5	
Inter-spinous (cm)	51.7	13.0	<0.0001		0.17	0.06	0.009		0.15	0.06	0.012		0.15	0.05	0.003	
Constant	–3019	1188	0.012	0.20	31.3	5.7	<0.0001	0.11	16.5	5.5	0.003	0.06	19.7	4.4	<0.0001	0.09
Gestational age (*w*)	136.3	23.8	<0.0001		0.55	0.11	<0.0001		0.40	0.11	<0.0001		0.38	0.09	<0.0001	
Maternal BMI (kg/m^2^)	–77.6	277.0	0.7		–2.44	1.33	0.068		–0.40	1.30	0.7		–0.51	1.04	0.6	
Inter-cristal (cm)	52.5	16.6	0.002		0.17	0.08	0.039		0.10	0.08	0.17		0.10	0.06	0.10	
Constant	–2580	1168	0.028	0.24	31.9	5.6	<0.0001	0.13	17.1	5.5	0.002	0.08	19.2	4.4	<0.0001	0.12
Gestational age (*w*)	128.2	23.3	<0.0001		0.53	0.11	<0.0001		0.38	0.11	0.001		0.38	0.09	<0.0001	
Maternal BMI (kg/m^2^)	–385.3	286.5	0.18		–3.59	1.38	0.01		–1.54	1.35	0.2		–1.35	1.08	0.2	
Conjugate (cm)	9.9	19.4	0.6		0.14	0.09	0.12		0.14	0.09	0.12		0.08	0.07	0.2	
Inter-spinous (cm)	43.8	15.7	0.006		0.08	0.08	0.2		0.08	0.07	0.2		0.10	0.06	0.079	
Inter-cristal (cm)	38.6	16.9	0.024		0.10	0.08	0.2		0.04	0.08	0.5		0.04	0.06	0.4	

Shading indicates predictors other than constant that are significant *P* < 0.05.

Beta, untransformed B-coefficient; SE, standard error of B-coefficient.

Maternal BMI natural log-transformed.

All models adjusted for birth order, by including (when significant *P* < 0.1) dummy variables for second-born and third+-born status.

Table 4 provides additional multiple regression models, testing whether associations of maternal pelvic dimensions with birth length or weight are mediated by maternal height or neonatal head girth. The association of maternal height with birth length was independent of pelvic dimensions, but associations of pelvic dimensions with birth length disappeared after adjusting for neonatal head girth, whereas those for maternal height remained. Maternal height was not a predictor of birth weight. Associations of pelvic dimensions with birth weight persisted, following adjustment for neonatal head girth. Maternal height was not a predictor of thorax girth. Associations of maternal pelvic dimensions with thorax girth disappeared after adjusting for head girth (data not shown).

**Table 4.** Multiple regression models of neonatal weight and length on maternal height and pelvic dimensions

Predictor	Neonatal length (cm)								Neonatal weight (g)							
	Beta	SE	*P*	*r*^2^	Beta	SE	*P*	*r*^2^	Beta	SE	*P*	*r*^2^	Beta	SE	*P*	*r*^2^
Constant	19.1	5.3	<0.0001	0.12	7.7	4.7	0.10	0.35	–3543	1140	0.002	0.15	–6196	939	<0.0001	0.46
Gestational age (*w*)	0.54	0.11	0.028		0.28	0.10	0.006		142.8	24.6	<0.0001		70.2	20.2	0.001	
Maternal height (cm)	0.05	0.02	<0.0001		0.04	0.02	0.035		6.9	5.0	0.16		4.9	4.0	0.2	
Neonatal head girth (cm)					0.66	0.07	<0.0001						169.2	14.7	<0.0001	
Constant	17.9	5.3	0.001	0.12	7.5	4.7	0.1	0.35	–4108	1121	0.001	0.19	–6524	931	<0.0001	0.47
Gestational age (*w)*	0.53	0.11	<0.0001		0.28	0.10	0.006		141.6	23.9	<0.0001		77.0	20.2	<0.0001	
Maternal height (cm)	0.04	0.02	0.071		0.04	0.02	0.049		3.1	5.0	0.5		2.7	4.0	0.5	
Conjugate (cm)	0.11	0.07	0.094		0.03	0.06	0.6		50.6	13.9	<0.0001		29.3	11.4	0.011	
Neonatal head girth (cm)					0.65	0.07	<0.0001						160.4	14.7	<0.0001	
Constant	18.2	5.3	0.001	0.13	7.6	4.7	0.11	0.35	–3995	1094	<0.0001	0.22	–6412	916	<0.0001	0.49
Gestational age (*w*)	0.51	0.11	<0.0001		0.28	0.10	0.007		130.6	23.6	<0.0001		71.7	20.0	0.001	
Maternal height (cm)	0.05	0.02	0.034		0.04	0.02	0.037		5.9	4.8	0.2		4.3	3.9	0.2	
Inter-spinous (cm)	0.11	0.05	0.048		0.03	0.05	0.5		53.7	11.5	<0.0001		33.3	9.5	0.001	
Neonatal head girth (cm)					0.65	0.07	<0.0001						156.5	14.6	<0.0001	
Constant	18.9	5.3	<0.0001	0.11	7.7	4.7	0.10	0.35	–3721	1106	0.001	0.20	–6244	913	<0.0001	0.49
Gestational age (*w*)	0.53	0.11	<0.0001		0.28	0.10	0.006		133.9	23.9	<0.0001		66.2	19.7	0.001	
Maternal height (cm)	0.05	0.02	0.041		0.04	0.02	0.036		4.0	4.9	0.4		2.8	3.9	0.4	
Inter-cristal (cm)	0.05	0.06	0.4		–0.00	0.05	0.9		47.8	12.2	<0.0001		36.3	9.8	<0.0001	
Neonatal head girth (cm)					0.66	0.07	<0.0001						163.2	14.3	<0.0001	

Shading indicates predictors other than constant that are significant *P* < 0.05.

Beta, untransformed B-coefficient; SE, standard error of B-coefficient.

Maternal BMI natural log-transformed.

All models adjusted for birth order, by including (when significant *P* < 0.1) dummy variables for second-born and third+-born status.

## DISCUSSION

Maternal BMI was strongly correlated with pelvic dimensions, whereas maternal height was more weakly associated with conjugate and inter-cristal diameters. Maternal height and BMI were each weak predictors of birth weight, but the only other neonatal outcome predicted by maternal size was length, predicted by maternal height. In contrast, maternal conjugate and inter-spinous diameters predicted all four components of neonatal size, while the inter-cristal diameter predicted birth weight and head girth. When these analyses were combined, pelvic dimensions displaced maternal BMI as a predictor of birth weight, explaining 11.6% of the variance and were associated with birth length independent of maternal height. Associations of pelvic dimensions with birth length or thorax girth were mediated by neonatal head girth, whereas for birth weight, pelvic dimensions remained predictors independent of head girth.

These findings indicate that in this population (a) maternal pelvic dimensions account for associations of maternal BMI with neonatal weight and this is independent of maternal height, and (b) head girth and body weight appear to be the key components of neonatal size associated with maternal pelvic dimensions.

In turn, these findings contribute to debate regarding the exact role of maternal/fetal nutrition in shaping long-term NCD risk, and the role of adaptation in this context. The finding that maternal BMI is strongly correlated with pelvic dimensions suggest that this component of maternal phenotype may not relate only to adiposity or lean mass, as widely assumed, but also to skeletal dimensions of relevance to fetal growth potential.

Exactly how plasticity in growth might be adaptive in early life, and yet also contribute to non-adaptive outcomes such as NCDs in later life, has remained controversial. The ‘predictive adaptive response’ hypothesis assumes that fetal growth patterns represent an adaptation to external ecological conditions, allowing phenotype to be tailored adaptively in anticipation of similar ecological conditions in later life [31]. An alternative ‘maternal capital’ hypothesis is that fetal growth is substantially (though not totally) buffered from external stimuli and stresses, and hence is strongly shaped by relatively stable components of maternal phenotype [32, 34], rather than external ecological factors. This is consistent with classic studies that identified inter-generational transmission of ‘maternal constraint’ through non-genetic mechanisms [25, 26], though it should be remembered that more plastic maternal traits such as smoking status and placental function as well as parity also contribute to birth weight variability within and between mothers.

Several components of maternal size and nutritional status are already well-recognized predictors of fetal growth variability [36, 54, 55]. Beyond these overall associations, we must also explain why offspring appear to *increase* in their sensitivity to maternal phenotype around the time of birth, as indicated by marked changes in the heritability of growth before and after birth. Fetal genes appear to ‘relax their control’ over growth before birth and then re-assert it after delivery (Fig. 1). This is associated with variability in infant growth, as small neonates tend to catch-up and large neonates catch-down [57].

Building on the pioneering work of Barker and colleagues, who linked flattened maternal pelvises with altered neonatal body proportions [47], we have argued previously that natural selection may have favoured such changes in growth regulation in order to reduce the likelihood of cephalo-pelvic disproportion, potentially fatal to fetus, mother or both [39]. Should a mother have experienced poor growth during her own development, her genes (marking her genetic potential) would no longer match her pelvic phenotype and this would also apply to those of her genes present in the fetus. The same scenario may explain why those genes that *do* impact birth weight typically have a small magnitude of effect [39].

The notion that the match between fetal size and maternal pelvic dimensions is subject to selective pressure is supported by research linking the risk of vaginal fistula, resulting from prolonged obstructed labour, with both maternal and offspring traits. In Ethiopia, for example, risk of fistula was greater in shorter women, and also in primiparous women, who may not have completed their pelvic growth. Complementary to that, risk of fistula was also increased if the fetus was male, attributable to their greater weight and head girth relative to female offspring [58]. Clearly, none of these traits is pathological, rather all represent normal components of population variability that can impact the relationship between maternal pelvic dimensions and the magnitude of fetal growth.

Variability in maternal pelvic dimensions may reflect both genotype and nutritional conditions through the maternal life-course. Inter-ethnic comparisons show that Indian mothers characterized by smaller average body size (height, BMI) produce larger neonates if the father is European (larger body size) rather than Indian [59], indicating a paternal influence on the magnitude of maternal nutritional investment during pregnancy. However, such mixed-ethnic pairings also show an elevated risk of cesarean delivery, whereas the contrasting union (European mother, Indian father) shows no such elevated risk [60]. Since birth weight is on average lower among Indian-father/European-mother pairings than among two European parents [59], the implication is that the magnitude of paternal demand for maternal investment in fetal growth has decreased across generations in the Indian population. Smaller pelvic dimensions among Indian mothers, raising the risk of cephalo-pelvic disproportion, may be an important mediating mechanism [39].

Through constraint generated by her pelvic dimensions, therefore, as well as via other mechanisms, a mother whose growth was suppressed during her own development may constrain the growth of her offspring during pregnancy, even if that offspring has the potential to grow large in post-natal life. This generates two independent risk factors for NCDs–poorer fetal growth, and rapid post-natal catch-up among those whose fetal growth constraint was greater relative to their growth potential. Poor fetal growth constrains organ growth and metabolic capacity, whereas catch-up growth elevates metabolic load [20]. Neither of these patterns of growth need relate strongly to maternal dietary intake or nutritional status during pregnancy, nonetheless they help understand the overall pathway to NCD risk. As highlighted by Leon and colleagues [61], it is those who have a low birth weight relative to their adult height–ie those whose late fetal growth was *suppressed*–who have elevated NCD risk.

The magnitudes of effect we report are modest, and our findings require replication in other studies. Furthermore, in this population we have shown that pelvic dimensions mediate only the association of maternal BMI, and not that of maternal height, with neonatal size. In other studies, both higher [42, 43] and lower [44] correlations of maternal height and pelvic dimensions have been reported. It is already known that correlations of maternal height with birth size are much stronger across populations [62] than within them [55]. It is possible, given inter-generational correlations in birth size [55], that the extent to which maternal pelvic dimensions shape fetal growth trajectory depends on the magnitude of any secular trend in height across generations. The maternal pelvis might exert stronger constraints on fetal growth when a downward secular trend is occurring, or when an upward trend is tailing off.

We have argued previously that in long-lived species such as humans, plasticity during fetal life may have less to do with ‘adapting’ to external ecological stresses than is often assumed [31], and more to do with matching growth and development to constraints generated by components of maternal phenotype that are stable, including those that responded to ecological conditions during her own development [21, 34]. In relation to the pelvis, this argument applies primarily to the second half of pregnancy, attributing elevated plasticity to the powerful selective pressure generated by cephalo-pelvic disproportion [39]. Fetal growth variability in early pregnancy also predicts NCD risk [7], but we can expect such associations to be less affected by maternal pelvic dimensions.

Once such elevated plasticity in late fetal life has evolved, it cannot prevent external events such as maternal famine during pregnancy, or factors influencing growth in early infancy, also leaving a long-term imprint on phenotype. Therefore, the selective pressures that favoured the enhancement of fetal plasticity in the past need not necessarily be those acting most strongly on it in contemporary populations. The specific role of maternal pelvic dimensions in the developmental origins of adult disease still requires elucidation.

More broadly, the key implication of our analysis is not that pelvic dimensions are necessarily strongly associated with fetal growth patterns in every mother-infant dyad; indeed populations appear to show substantial variability in the magnitude of this association, as discussed above. Rather, the important implication is that the *threat* of cephalo-pelvic disproportion may have selected for greater physiological sensitivity to maternal phenotype in late pregnancy, resulting in a developmental period characterized by high plasticity that is not driven primarily by benefits of adapting to *external* ecological conditions. Further work on this issue may benefit from cross-species comparisons, to understand whether the magnitude of offspring plasticity before and after birth differs according to whether delivery does or does not face the kind of constraint generated by maternal pelvic dimensions.

**Conflict of interest**: None declared.
